# Antibiotic resistance, pathogenicity and genomic characteristics of a novel sequence type 6839 *Klebsiella pneumoniae* strain isolated from cultured bullfrogs (*Rana catesbeiana*)

**DOI:** 10.3389/fcimb.2026.1864334

**Published:** 2026-09-04

**Authors:** Yafeng Dou, Haochen Wang, Qikai Chen, Bo Zhang, Decheng Wang, Xuan Xia, Feng Fan, Zhihao Wu, Jihong Li

**Affiliations:** 1 Department of Pathology, The Second People’s Hospital of China Three Gorges University, Yichang, China; 2 College of Medical Science, Three Gorges University, Yichang, China; 3 Hubei Key Laboratory of Tumor Microenvironment and Immunotherapy, China Three Gorges University, Yichang, China; 4 Institution of Infection and Inflammation, China Three Gorges University, Yichang, China; 5 State Key Laboratory of Agricultural Microbiology, Huazhong Agricultural University College of Fisheries, Wuhan, China

**Keywords:** American bullfrog, *Klebsiella pneumoniae*, multidrug-resistant, pathogenicity, whole-genome sequence

## Abstract

**Introduction:**

Klebsiella pneumoniae is the most frequently encountered multidrug-resistant bacterial pathogen in humans, but its pathogenic effects on aquatic animals are not well documented. *K. pneumoniae* has recently been identified as a significant cause of death and illness in American bullfrogs (*Rana catesbeiana*). In the present study, the dominant bacteria were isolated from a diseased bullfrog with neurological signs in Hanchuan, Hubei Province, China.

**Methods:**

The ZYRC75 isolate was identified by Gram staining, morphological observations, biochemical assays and 16S rRNA sequencing. Antimicrobial susceptibility testing and artificial infection testing were conducted to determine its resistance phenotype and pathogenicity. The whole genome of the ZYRC75 strain was also sequenced.

**Results:**

The isolate ZYRC75 was identified as K. pneumoniae and assigned to ST6839, a novel sequence type. It displayed multidrug resistance against penicillins (carbenicillin, piperacillin, ampicillin, penicillin), chloramphenicol, trimethoprim and tetracycline. Infection experiments confirmed the isolate exhibited significant pathogenicity in bullfrogs. ZYRC75 possessed a single circular chromosome of 5,454,922 bp, carrying 179 virulence‑related genes and 157 antimicrobial‑resistance genes.

**Discussion:**

This investigation presents a detailed phenotypic and genomic profile of ZYRC75, revealing its antimicrobial resistance and virulence, and establishing a basis for future studies on the pathogenicity of *K. pneumoniae*.

## 1 Introduction

*Klebsiella pneumoniae*, a Gram-negative bacterium belonging to the Enterobacteriaceae family, was first identified in 1882 (Paczosa and Mecsas, 2016). It is commonly found in various environments like soil and water and is known to cause numerous human infections, including as neonatal sepsis, hospital-acquired pneumonia, liver abscesses and chronic intestinal diseases (Lam et al., 2018; Martin and Bachman, 2018; Lam et al., 2021). Beyond its clinical significance, this pathogen exhibits a broad ecological distribution, colonizing diverse environmental niches and host species (Bengoechea and Sa Pessoa, 2019). This bacterium leads to liver abscesses in nonhuman primates (Twenhafel et al., 2008), subclinical mastitis in dairy cows (Cheng et al., 2021), and respiratory infections in cats and dogs (Silva et al., 2018; Silva et al., 2022). *K. pneumoniae* has been identified as a pathogen in aquatic animals, causing pleuritis and suppurative bronchopneumonia in sea lions (Jang et al., 2010), liver and kidney necrosis with peritoneal hemorrhage in carp (Das et al., 2018), and edematous bodies with hyperemia in leeches (Yibin et al., 2021). *K. pneumoniae* has recently emerged as a cause of fatal dermatosepticemia in amphibians, with significant outbreaks causing high mortality in species such as the brown tree frog (*Litoria ewingii*) (SChadich and Cole, 2010), the black-spotted frogs (*Pelophylax nigromaculatus*) (Li et al., 2023) and the American bullfrog (*Rana catesbeiana*) (Lin et al., 2023). Its increasing prevalence and clinical impact in frog populations represent a growing threat.

The American bullfrog (*Rana catesbeiana*), originally native to North America and introduced to China during the 1950s, has been subject to intensive aquaculture practices since the 1990s (Lin et al., 2023). This species holds significant commercial value, prompting a rapid expansion of aquaculture operations to satisfy increasing market demands (Zhang et al., 2015). Initially introduced to China from Cuba and Japan for consumption purposes (Wu et al., 2004), bullfrog farming has experienced substantial growth and has been established in numerous provinces across the country (Zhang et al., 2015). In recent years, the prevalence of diseases affecting bullfrogs has increased, with bacterial outbreaks like epidemic meningitis-like disease and red leg syndrome posing significant challenges to the industry (Pasteris et al., 2006; Trimpert et al., 2021). Epidemic meningitis-like disease, in particular, has been frequently reported, with clinical manifestations in bullfrogs including torticollis, unilateral head tilting, circling behavior, and anorexia; mortality rates range from 60% to 90% within days to weeks following onset (Wei et al., 2023). Research indicates that bacterial diseases are the primary contributors to substantial losses in commercial bullfrog farming (Mauel et al., 2002; An et al., 2024). Consequently, to ensure the sustainable and robust development of the frog farming industry, further research into bacterial diseases in aquaculture is imperative.

In this study, we isolated, purified, and cultured the pathogenic isolate ZYRC75 from diseased cultured bullfrogs in Hanchuan City, Hubei Province, China during September 2023. The pathogenic isolate in the sick bullfrogs was identified as *K. pneumoniae* through growth, biochemical, and phylogenetic analyses, with its pathogenicity confirmed via artificial infection experiments in bullfrogs. We also applied the whole genome sequence to analyze the genomic characteristics, antimicrobial susceptibility and virulence factor profiles in bullfrog *K. pneumoniae* isolate. Our research provides a scientific basis for future investigations into its pathogenic mechanisms, antimicrobial resistance characteristics of *K. pneumoniae*.

## 2 Materials and methods

### 2.1 Bullfrogs and tissue sampling

A total of 60 healthy bullfrogs, weighing between 40 and 60 grams, were sourced from a farm in Hanchuan City, Hubei Province, China. They were maintained in aerated water tanks at 28 ± 1°C and fed daily. The experiments complied with the Animal Ethics Committee regulations at Huazhong Agricultural University.

### 2.2 Experimental animals

Bullfrogs for the infection experiment were sourced from a breeding farm in Hanchuan, Hubei Province. All of the experimental animals were artificially reared in the indoor freshwater recirculating system. An air stone was installed in each tank to ensure sufficient dissolved oxygen levels. The experiment maintained a photoperiod mimicking natural daylight cycles, with water temperature precisely controlled at 28 °C. Fish were fed commercial floating dry pellets twice daily until satiation. To preserve water quality and prevent contamination, siphoning techniques were employed to remove any leftover food or waste from the tank before feeding. The experiments followed the guidelines of Huazhong Agricultural University (HZAUFI‐2024‐0026).

### 2.3 Bacterial isolation and purification

Diseased bullfrogs were anesthetized with an overdose of MS-222 and euthanized by spinal cord transection. Bacterial isolates from the brain, kidney, spleen, and liver were initially cultured on Luria-Bertani (LB) agar plates at 28 °C for 24–30 hours. Colonies were then transferred to new LB agar plates for a further 20-hour incubation. Single colonies were inoculated into 5 mL of LB broth and cultured at 28 °C with shaking at 170 rpm for 24 hours. The bacterial cells were resuspended in LB broth with 20% sterile glycerol and stored at −80 °C.Colony size and morphological characteristics were assessed by Gram staining and examined under light microscopy.

### 2.4 Molecular identification

The bacteria were cultured on an LB plate at 28 °C for 24 hours. A single colony was used as a template for PCR amplification in 20 μL of sterile water. The 16S rDNA gene was amplified with primers 27 F (5′-AGAGTTTGATCTGGCTCAG) and 1492 R (5′-GGTTACCTTGTTACGACTT) (Yibin et al., 2021). The PCR reaction mixture consisted of 1 μL template, 1 μL of each primer, 25 μL of 2 × Ex Tap mix, and 22 μL of ddH2O.PCR was conducted with an initial denaturation at 95 °C for 3 minutes, followed by 38 cycles of 98 °C for 10 seconds, 60 °C for 30 seconds, and 72 °C for 2 minutes, concluding with a final extension at 72 °C for 7 minutes. The primers were synthesized by Tsingke in Wuhan, China. PCR products underwent agarose gel electrophoresis and were sequenced by Tsingke in Wuhan, China. The sequence was analyzed using BLAST against the GenBank database. A phylogenetic tree was constructed using the agglomerative method in MEGA 5.1 software, and the tree was validated using the bootstrap method 1,000 times.

### 2.5 Phenotypic characterization

Colony morphology and Gram staining were used to initially assess the bacterial isolate. Afterward, a commercial micro-biochemical identification system was employed for biochemical profiling (Qingdao Hope Bio-Technology Co.), following the manufacturer’s guidelines. Following a 48-hour incubation period at 28 °C, a skilled microbiologist interpreted the findings.

### 2.6 Antibiotic susceptibility tests

A group of 39 antibiotics, including polypeptides: polymyxin B (300 μg); sulfonamides: compound-sulfamethoxazole (23.75 μg/1.25 μg), sulfafurazole (300 μg); dihydrofolate reductase inhibitor: trimethoprim (5 μg); tetracyclines: doxycycline (30 μg), minocycline (30 μg), tetracycline (30 μg); rifamycins: rifampicin (5 μg); lincosamides: clindamycin (2 μg); nitrofurans: nitrofurantoin (300 μg); macrolides: midecamycin (30 μg), erythromycin (15 μg), clarithromycin(15 μg); carbapenems: imipenem (10 μg), meropenem (10 μg); quinolones: levofloxacin (5 μg), ofloxacin (5 μg), norfloxacin (10 μg), ciprofloxacin (5 μg), enrofloxacin (10 μg); cephalosporins: ceftazidime (30 μg), cefoperazone (75 μg), cephalothin (30 μg), cefuroxime (30 μg), ceftriaxone (30 μg), cefazolin (30 μg); aminoglycosides: tobramycin (30 μg), neomycin (30 μg), kanamycin (30 μg), streptomycin (10 μg), amikacin (30 μg), gentamicin (10 μg), spectinomycin (100 μg); penicillins: carbenicillin (100 μg), piperacillin (100 μg), ampicillin (10 μg), penicillin (10 μg); chloramphenicols: chloramphenicol (30 μg), florfenicol (30 μg), were used for antimicrobial susceptibility testing.

According to the protocol proposed by the Clinical and Laboratory Standards Institute (CLSI) M100-Ed35–2025 (CLSI, 2025), the Kirby–Bauer method was used to identify the drug resistance profile of the isolated strain (Bauer et al., 1966). Briefly, the isolated bacteria were cultured in MHB (cation-adjusted Mueller–Hinton broth) and kept at 30 °C on a shaker for 12 hours. Log-phase bacterial cultures were adjusted to a 0.5 McFarland unit with normal saline and 100 μL of the bacterium at a concentration of 1×10^9^ CFU/mL was applied to the surface of MHA (cation-adjusted Mueller–Hinton agar). Standard antibiotic disks (Hangzhou Microbial Reagent Co., Ltd., China) were positioned on the surface of the MHA and incubated at 35 °C for 24 hours to determine the inhibition zone diameter. The results were labeled “sensitive,” “intermediately sensitive,” and “drug-resistant”. *Escherichia coli* (ATCC 25922) and *K. pneumoniae* (ATCC 700603) were used as the quality control strains for antimicrobial susceptibility testing.

### 2.7 Experimental infection test

Challenge experiments were conducted on healthy bullfrogs to assess the virulence of the isolate ZYRC75. Bullfrogs were divided into six groups, with five groups being challenged and one serving as a negative control. The isolate ZYRC75 was cultured to logarithmic growth phase. The bacterial concentration was determined by plate counts through diluting 100 times continuously. After washing twice with PBS, the bacteria were resuspended in PBS to final doses of 1 × 10^7^, 1 × 10^6^, 1 × 10^5^, 1 × 10^4^, and 1 × 10^3^ CFU in 200 μL, respectively. Ten bullfrogs in each group were administered the corresponding doses of bacteria via intramuscular injection and were observed for 14 days following infection. The LD_50_ value was determined using the Karber’s method (Kärber, 1931). The steps for calculating the LD_50_ are listed below.

1. Core Formula: LD_50_= lg^-1^[X_m_ − i(∑p−0.5)]
2. Symbol Definition:
  X_m_: Log value of the highest fully lethal dose.
  i: Logarithmic interval between two adjacent doses.
  ∑p: Total sum of mortality proportions.

### 2.8 Histopathological observation

For histopathological examination in pathogenicity studies, bullfrog tissue samples from the liver, spleen, kidneys, intestines, and brain were fixed in 10% buffered formalin, trimmed, dehydrated with ethanol, and embedded in paraffin blocks. The sections were stained with Mayer’s hematoxylin and eosin (H&E) and examined under a light microscope.

### 2.9 Whole genome sequence and analysis

Strain ZYRC75 was grown in LB medium at 28 °C for 24 hours and then collected by centrifuging at 10,000 g for 5 minutes. The extraction of total genomic DNA was conducted following previously established protocols, and whole-genome sequencing was executed utilizing the Illumina NovaSeq6000 sequencing platform. Personal Biotechnology Co., Ltd (Shanghai, China) facilitated the sequencing services. Genome assembly and subsequent polishing were performed using SPAdes version 3.15.5 and Pilon version 2.2, respectively. The full genome sequence of strain ZYRC75 is accessible in NCBI GenBank under biosample accession SAMN57421799 and ID PRJNA1456768 (https://www.ncbi.nlm.nih.gov/biosample/?term=SAMN57421799).

Circular genome maps for each isolate were generated employing Circos software. The genome analysis utilized various software tools: Glimmer 3.02 and GeneMarkS for coding sequences (CDSs), tRNAscan-SE for transfer RNA (tRNA), Barrnap for ribosomal RNA (rRNA), Tandem Repeats Finder for tandem repeats, IslandPath-DIMOB for gene islands (GIs), Phigaro for prophages, Minced for CRISPR-Cas systems, Integron Finder for integrons, and ISEScan for insertion sequences (ISs). The KEGG database (Kanehisa and Sato, 2020), was used for annotating predicted CDSs, and virulence-related and antimicrobial resistance genes were identified using the Virulence Factors Database (VFDB) and the Comprehensive Antibiotic Resistance Database (CARD) as previously described (Cao et al., 2022).

### 2.10 Multi-locus sequence typing

Through whole-genome sequencing, the MLST software (version 2.23.0) was employed to screen the assembled genome sequences of the isolate for overlapping regions of seven conserved housekeeping genes (*gapA*, *infB*, *mdh*, *pgi*, *phoE*, *rpoB*, *tonB*). Based on the allelic profiles of these seven genes, the corresponding sequence type was assigned to the isolate. The identified ST was queried and validated using the *Klebsiella* PasteurMLST database (https://bigsdb.pasteur.fr/cgi-bin/bigsdb/bigsdb.pl?db=pubmlst_*klebsiella*_isolates).

### 2.11 Statistical analysis

Kaplan-Meier plots were generated using Graphpad Prism v11 (GraphPad Software, San Diego, CA, USA). The log-rank (Mantel-Cox) test was used to calculate a *P*-value for overall infected bullfrog survival.

## 3 Results

### 3.1 Clinical signs of natural infection

**Figure 1:**
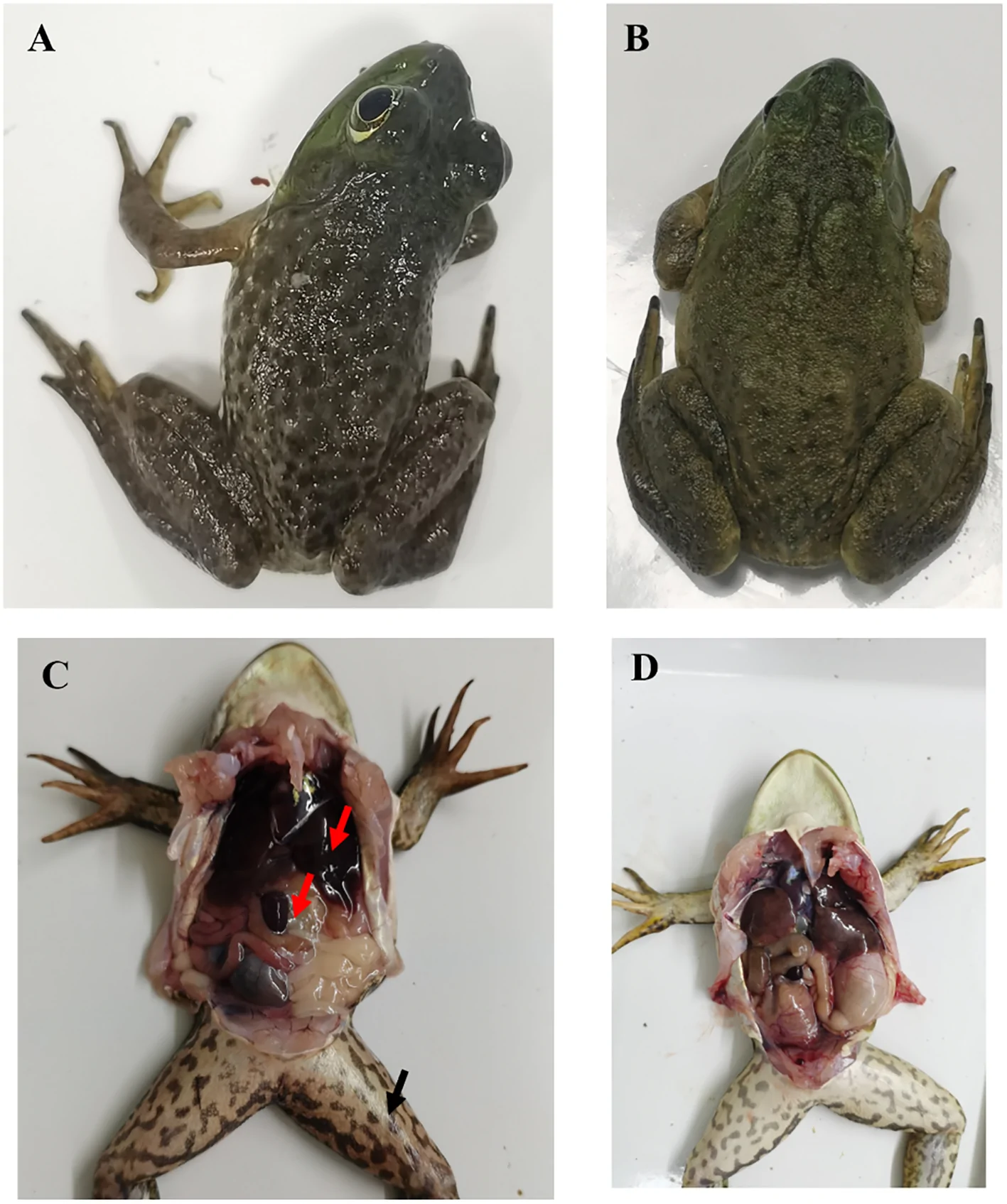
The clinical symptoms of bullfrogs infected with *K. pneumoniae*. **(A, C)** infected bullfrog, **(A)** Head biased to one side; **(B)** Arrows indicate bleeding skin (black arrow) and rotten fins and enlarged liver (red arrow) and spleen (red arrow). **(B, D)** control bullfrog.

The sick bullfrogs exhibited bent heads, rigid bodies, and movement disorders (**Figure 1A**), along with red patches on their abdomen and legs (**Figure 1C**), compared with healthy bullfrogs in the control group (**Figures 1B, D**). Additionally, organs like the spleen, kidney, and liver were swollen (**Figure 1C**).

### 3.2 Isolation and identification of pathogenic bacteria

**Figure 2:**
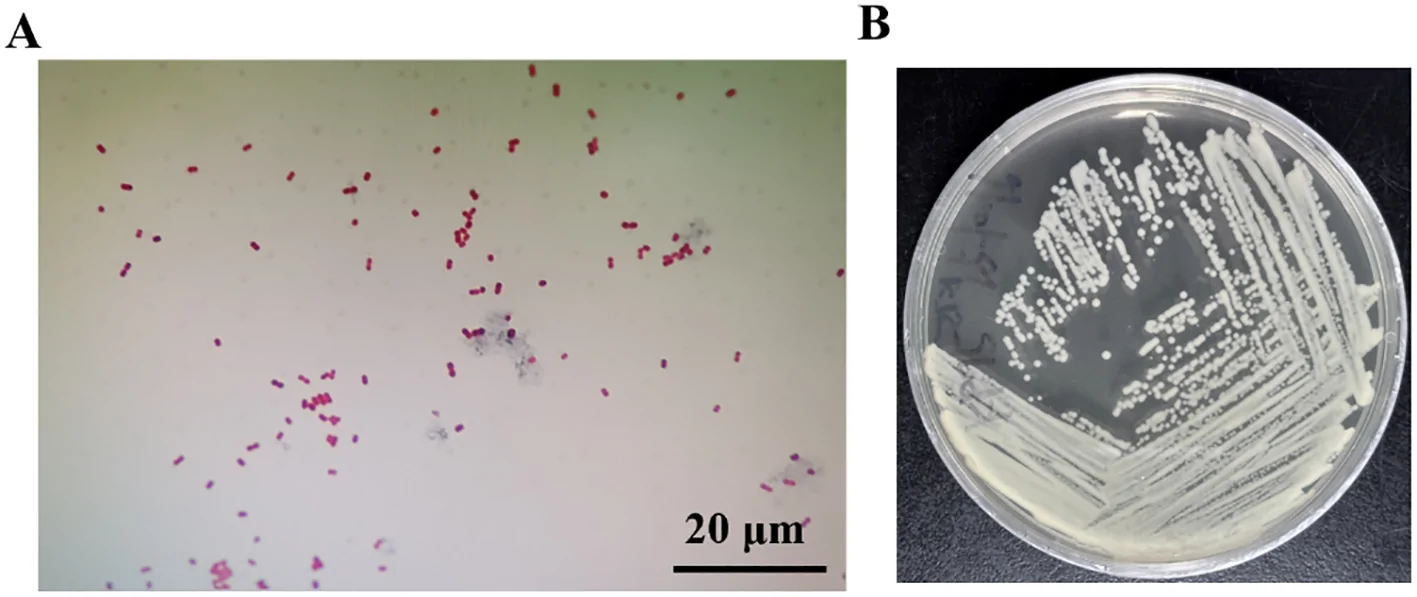
Morphological characteristics of the isolated strain. **(A)** Gram’s stain microscopic results of isolated strain. **(B)** LB agar.

**Figure 3:**
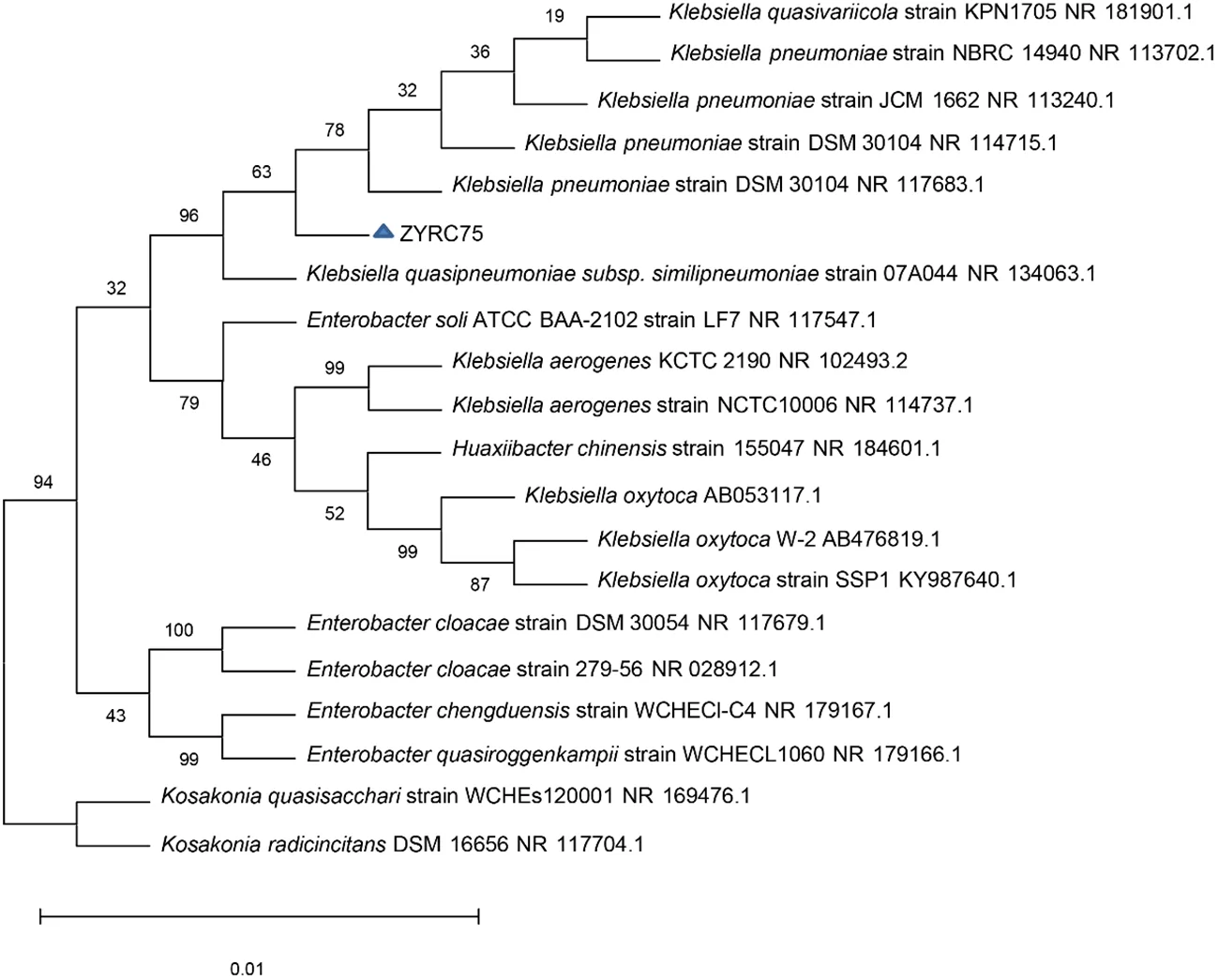
Phylogenetic tree constructed based on the 16S rRNA gene sequence of isolated strain. The serial number in parentheses is the GenBank accession number of the relevant strain; the number on the branching point is the support rate of bootstrap; Bar=0.01 is nucleotide divergence.

The Gram stain revealed a typical oval or short rod-shaped form (**Figure 2A**), confirming the isolate as Gram-negative. The colonies of bacteria on LB agar plates were smooth, circular, and greyish-white (**Figure 2B**). The 16S rRNA gene sequence of ZYRC75 was identified and uploaded to the GenBank database (accession no. PZ299193). The NCBI nucleotide database BLAST analysis revealed that this sequence had the highest resemblance to *K. pneumoniae*. The neighbor-joining (NJ) method was employed to construct a phylogenetic tree using 16S rRNA gene sequences from members of the *Klebsiella* genus. The findings indicated that isolate ZYRC75 grouped with *K. pneumoniae* strains and exhibited the greatest similarity to *K. pneumoniae* strain NR_117683.1 (**Figure 3**).

### 3.3 Biochemical analysis and antimicrobial susceptibility testing

A series of biochemical assays were performed. The results demonstrated that strain ZYRC75 fermented glucose, lactose, and sucrose, and exhibited urease and lysine decarboxylase activities. The strain tested positive for Voges–Proskauer, D-mannitol, D-sorbitol, citrate utilization, and malonate utilization, but negative for methyl red, arginine dihydrolase, and ornithine decarboxylase. Furthermore, strain ZYRC75 did not produce hydrogen sulfide (H_2_S) (**Table 1**).

**Table 1:** Phenotypic characterization of the isolate.

Test item	ZYRC75	*K. pneumoniae* KPY01 (Rastegar et al., 2019)
Methyl red	–	–
Voges-Prokaver	+	+
Lactose	+	+
Glucose	+	+
Sucrose	+	+
D-Mannitol	+	+
D-sorbitol	+	+
Citrate	+	+
H_2_S	–	–
Urease	+	+
Arginine dihydrolase	–	–
Lysine decarboxylase	+	+
Ornithine decarboxylase	–	–
Malonate	+	+

“+” = positive reaction; “-” = negative reaction.

Antimicrobial susceptibility testing was performed against 39 antimicrobial agents using the Kirby–Bauer disk diffusion method. The ZYRC75 isolate was only susceptible to polymyxins (polymyxin B), but resistant to penicillins (carbenicillin, piperacillin, ampicillin, penicillin), carbapenems (imipenem, meropenem), chloramphenicol, dihydrofolate reductase inhibitors (trimethoprim), and tetracyclines (tetracycline). The nitrofuran derivative nitrofurantoin and sulfonamide derivative compound sulfamethoxazole exhibited intermediate susceptibility to strain ZYRC75 (**Table 2**).

**Table 2:** Results of drug susceptibility testing.

Drugs class	Drugs names	Judgment standard of inhibition zone diam (mm)			Dose (μg/disc)	Inhibition zone diameter (mm)	Sensitivity	
		Resistant	Intermediately sensitive	Sensitive		35°C		
Polypeptides	Polymyxin B	≤7	8-11	≥12	300	17	S	
Sulfonamides	Compound Sulfamethoxazole	≤10	11-15	≥16	23.75 /1.25	11	R	
	Sulfafurazole	≤12	13-16	≥17	300	10	R	
Dihydrofolate Reductase Inhibitor	Trimethoprim	≤10	11-15	≥16	5	10	R	
Tetracyclines	Doxycycline	≤15	16-18	≥19	30	13	R	
	Minocycline	≤15	16-18	≥19	30	12	R	
	Tetracycline	≤15	16-18	≥19	30	14	R	
Rifamycins	Rifampicin	≤16	17-19	≥20	5	12	R	
Lincosamides	Clindamycin	≤14	15-20	≥21	2	12	R	
Nitrofurans	Nitrofurantoin	≤14	15-16	≥17	300	16	I	
Macrolides	Erythromycin	≤13	14-22	≥13	15	11	R	
	Midecamycin	≤15	16-18	≥19	30	12	R	
	Clarithromycin	≤13	14-17	≥18	15	11	R	
Carbapenems	Imipenem	≤19	20-22	≥23	10	18	S	
	Meropenem	≤19	20-22	≥23	10	16	S	
Quinolones	Levofloxacin	≤15	16-18	≥19	5	13	R	
	Ofloxacin	≤15	16-18	≥19	5	14	R	
	Norfloxacin	≤12	13-16	≥17	10	11	R	
	Ciprofloxacin	≤15	16-20	≥21	5	13	R	
	Enrofloxacin	≤16	17-22	≥23	10	15	R	
Cephalosporins	Ceftazidime	≤17	18-20	≥21	30	13	S	
	Cefoperazone	≤17	18-20	≥21	75	16	R	
	Cephalothin	≤14	15-17	≥18	30	12	R	
	Cefuroxime	≤14	15-17	≥18	30	11	R	
	Ceftriaxone	≤22	23-25	≥26	30	17	R	
	Cefazolin	≤19	20-22	≥23	30	17	R	
Aminoglycosides	Tobramycin	≤12	13-14	≥15	10	10	R	
	Amikacin	≤14	15-17	≥18	30	12	R	
	Gentamicin	≤12	13-16	≥17	10	8	R	
	Kanamycin	≤14	15-17	≥18	30	10	R	
	Spectinomycin	≤14	15-17	≥18	100	11	R	
	Neomycin	≤12	13-16	≥17	30	10	R	
	Streptomycin	≤11	12-14	≥15	10	8	R	
Penicillins	Carbenicillin	≤19	20-22	≥23	100	12	R	
	Piperacillin	≤16	16-64	≥64	100	6	R	
	Ampicillin	≤13	14-16	≥17	10	9	R	
	Penicillin	≤17	18-20	≥21	10	16	R	
Chloramphenicols	Chloramphenicol	≤12	13-17	≥18	30	10	R	
	Florfenicol	≤14	15-18	≥19	30	13	R	

### 3.4 Experimentally infected bullfrogs

**Figure 4:**
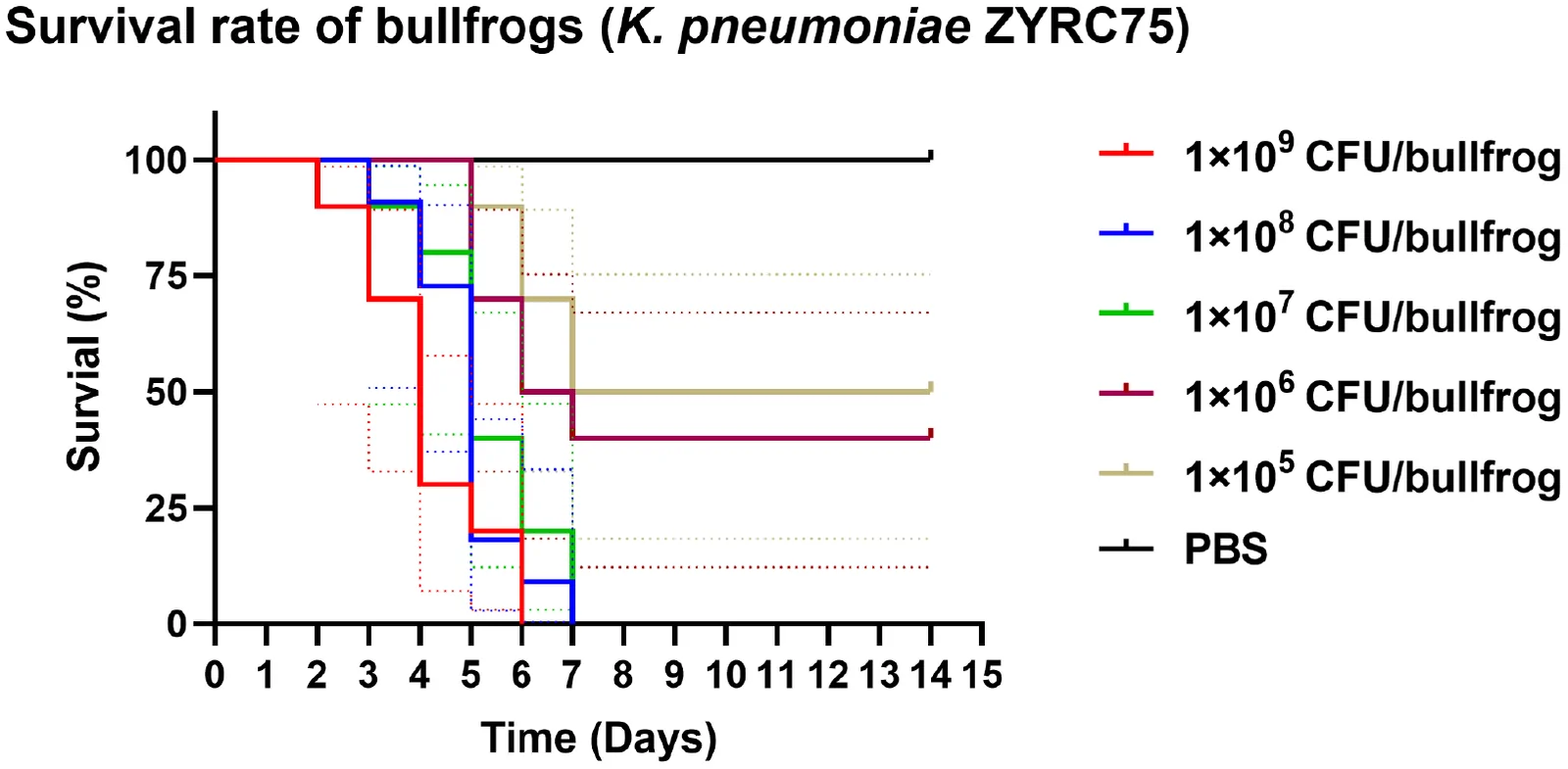
Results of the pathogenicity assessment of *K. pneumoniae* ZYRC75 at different levels in bullfrogs. Kaplan-Meier survival analysis of infected bullfrogs at doses ranging from 1×10^9^ CFU/bullfrog to 1×10^5^ CFU/bullfrog. After 14 days, the survival rates of bullfrogs infected with 1×10^9^ CFU/bullfrog, 1×10^8^ CFU/bullfrog and 1×10^7^ CFU/bullfrog were all lower than those infected with 1×10^6^ CFU/bullfrog and 1×10^5^ CFU/bullfrog (1×10^7^ CFU/bullfrog *vs.* 1×10^6^ CFU/bullfrog, *P* = 0.0275; 1×10^7^ CFU/bullfrog *vs.* 1×10^5^ CFU/bullfrog, *P* = 0.0031; 1×10^8^ CFU/bullfrog *vs.* 1×10^6^ CFU/bullfrog, *P* = 0.0047; 1×10^8^ CFU/bullfrog *vs.* 1×10^5^ CFU/bullfrog, *P* = 0.0005; 1×10^9^ CFU/bullfrog *vs.* 1×10^6^ CFU/bullfrog, *P* = 0.0012; 1×10^9^ CFU/bullfrog *vs.* 1×10^5^ CFU/bullfrog, *P* = 0.0002).

Bullfrogs challenged with the highest bacterial dose (1×10^9^ CFU/bullfrog) exhibited abdominal distension and lethargy 2 days post-artificial infection, with one case of acute death. Mortality rates increased significantly between 3 and 7 days post-infection of 1 × 10^9^, 1 × 10^8^, 1 × 10^7^, 1 × 10^6^ and 1 × 10^5^ CFU/bullfrog. On the 14th day after infection, the cumulative mortality rates were 100%, 100%, 100%, 60%, and 50%, respectively (**Figure 4**). During the observation period, the dead and dying bullfrogs were all counted and recorded, leading to an LD_50_ calculation of 2.51 × 10^3^ CFU/bullfrog in this study. There were no deaths or visible abnormal alterations observed in the control group. The post-mortem analysis of bullfrogs infected experimentally revealed organ swelling due to congestion, consistent with naturally infected bullfrogs. Additionally, *K. pneumoniae* was successfully separated and confirmed from all deceased infected bullfrogs.

### 3.5 Histopathological analysis

**Figure 5:**
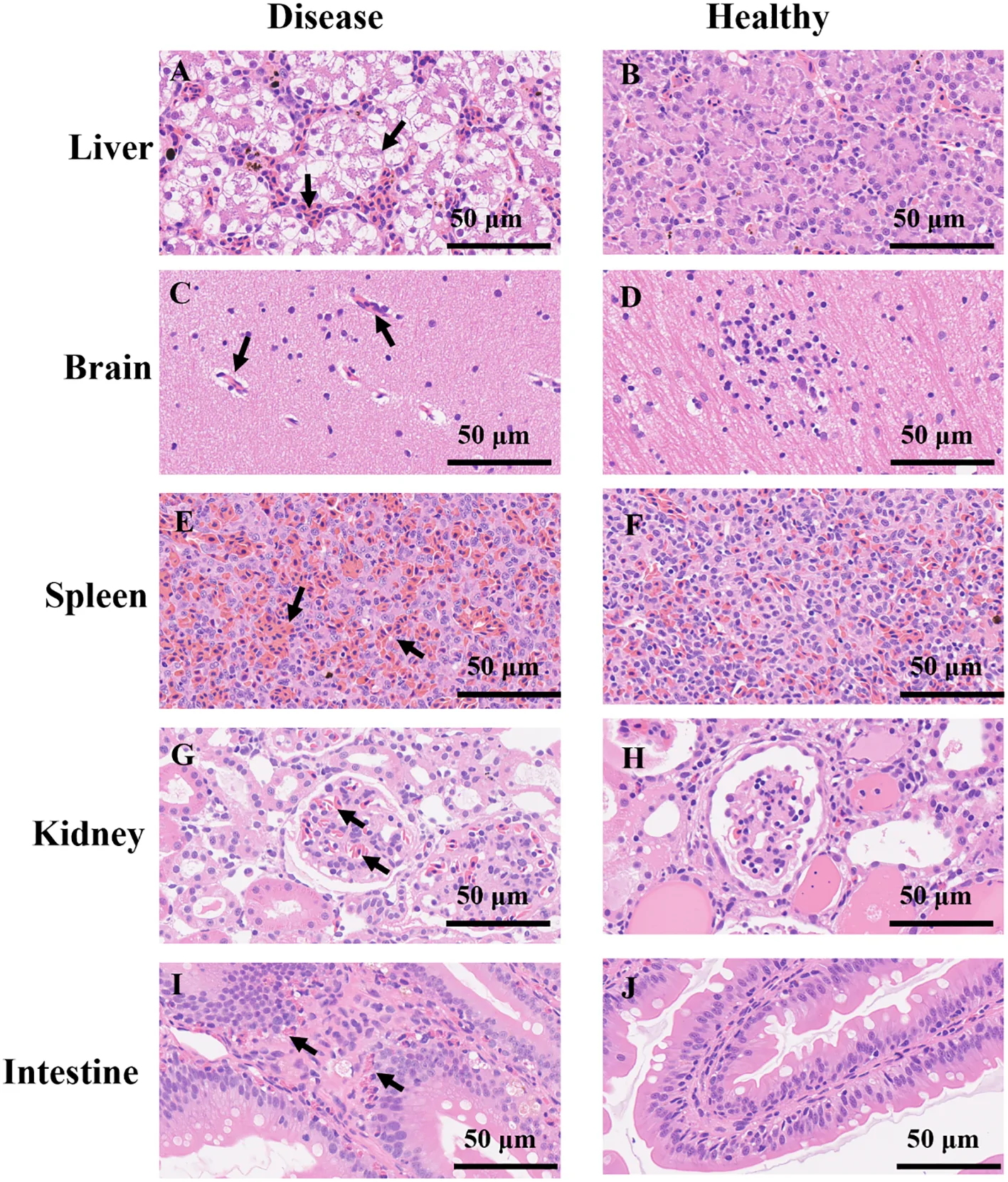
Histopathological observation of organs in bullfrogs infected with *K. pneumoniae* ZYRC75. The histologically affected liver **(A)**, spleen **(E)**, kidney **(G)**, intestine **(I)**, and brain **(C)** of bullfrogs were observed alongside healthy tissue sections of liver **(B)**, spleen **(F)**, kidney **(H)**, intestine **(J)**, and brain **(D)**, where the prominent indicators of injury to the brain, spleen, kidney, and intestine are hemorrhage (black arrow), while the liver shows hemorrhage and hyaline degeneration as the most evident signs of damage.

Pathologically, histological changes were observed in the livers (**Figure 5A**), brains (**Figure 5C**), spleens (**Figure 5E**), kidneys (**Figure 5G**) and intestines (**Figure 5I**) of diseased bullfrogs. Compared with the livers (**Figure 5B**), brain (**Figure 5D**), spleen (**Figure 5F**), kidney (**Figure 5H**) and intestine (**Figure 5J**) of healthy bullfrogs. The most prominent signs of damage in the brain, spleen, kidney and intestine were hemorrhage (black arrows), while the liver exhibited hemorrhage and hyalinization as the most evident signs of damage. The results indicate that the *K. pneumoniae* isolate ZYRC75 can cause multi-organ damage.

### 3.6 Whole genome sequencing and characterization of *K. pneumoniae* strain ZYRC75

**Figure 6:**
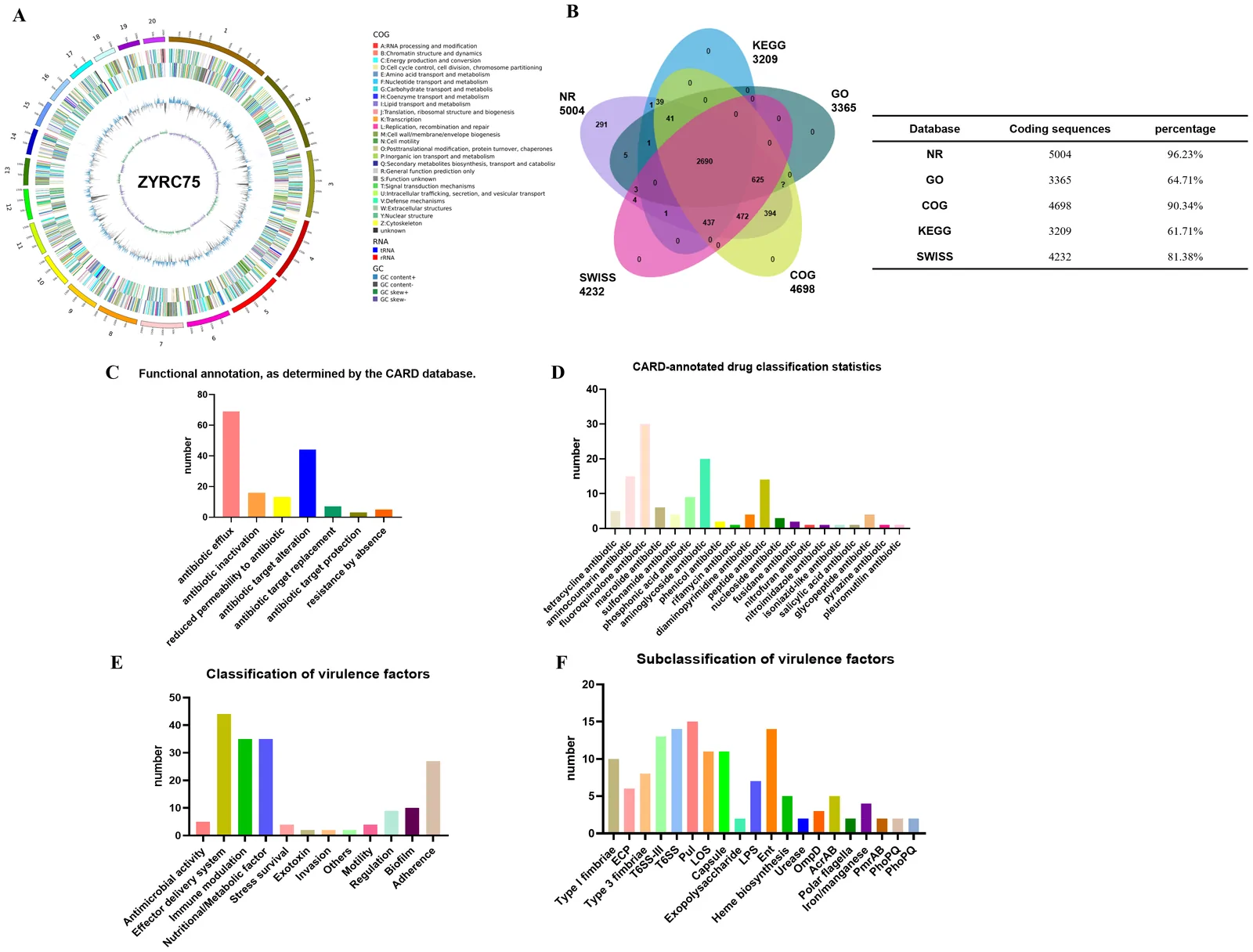
Whole genome atlas of *K. pneumoniae* ZYRC75. **(A)** Genomic mapping of the ZYRC75 strain. **(B)** Venn diagram illustrating the annotation outcomes across all gene function databases. **(C)** Statistics on drug classification annotated by CARD. **(D)** Functional annotation based on the CARD database. **(E)** Classification of virulence factors according to the VFBD database. **(F)** Subclassification of virulence factors, as determined by the VFBD database.

Whole genome sequencing was performed to better understand the pathogenesis of strain ZYRC75. This strain had a genome consisting of 5,454,922 base pairs and a GC content of 57.20% (**Figure 6A**). The genome comprises 5200 open reading frames (ORFs) with an average length of 908 base pairs, accounting for 86.62% of the total genome. The analysis of non-coding RNA (ncRNA) revealed 63 transfer RNAs (tRNAs) spanning 4,948 bp, a 16S rRNA of 1,536 bp, and a 23S rRNA of 2,899 bp. ZYRC75 was designated as ST6839, a novel type, based on sequence analysis of seven housekeeping genes and allelic profile in the *K. pneumoniae* MLST web database (**Table 3**). The ZYRC75 genome’s 5200 coding sequences were analyzed across the Non-Redundant Protein Sequence Database (NR), Kyoto Encyclopedia of Genes and Genomes (KEGG), Gene Ontology (GO), Clusters of Orthologous Groups of proteins (COG), and Swiss databases to acquire functional annotations. This analysis identified 2690 proteins with predicted functions consistent across all five databases (**Figure 6B**). Among the 5200 proteins encoded by ORF, 5182 were annotated, which accounts for 99.65%, and 86.62% were consistent with *K. pneumoniae* proteins. BLAST analysis using the Comprehensive Antibiotic Resistance Database (CARD) identified 157 antibiotic resistance genes, predominantly associated with cephalosporin antibiotics, followed by aminocoumarin, peptide, and aminoglycoside antibiotics (**Figure 6D**). According to the CARD database, resistance mechanisms primarily involved antibiotic efflux and modifications of antibiotic targets (**Figure 6C**). The primary virulence factors identified through BLASTx analysis of the ZYRC75 genome sequencing data with protein sequences from the VFDB were the Effector delivery system, Immune modulation factors, and Nutritional/Metabolic factor (**Figure 6E**). Among the factors for the Effector delivery system, T6SS genes were the most numerous with 14 genes, followed by 13 genes related to T6SS-III. In terms of genes linked to Immune modulation, 11 genes were tied to Capsule and 11 were linked to the LOS (**Figure 6F**).

**Table 3:** ST type of the isolated strain.

MLST housekeeping gene number							ST
gapA	infB	mdh	pgi	phoE	rpoB	tonB	
4	4	485	1	7	4	111	6839

**Figure 7:**
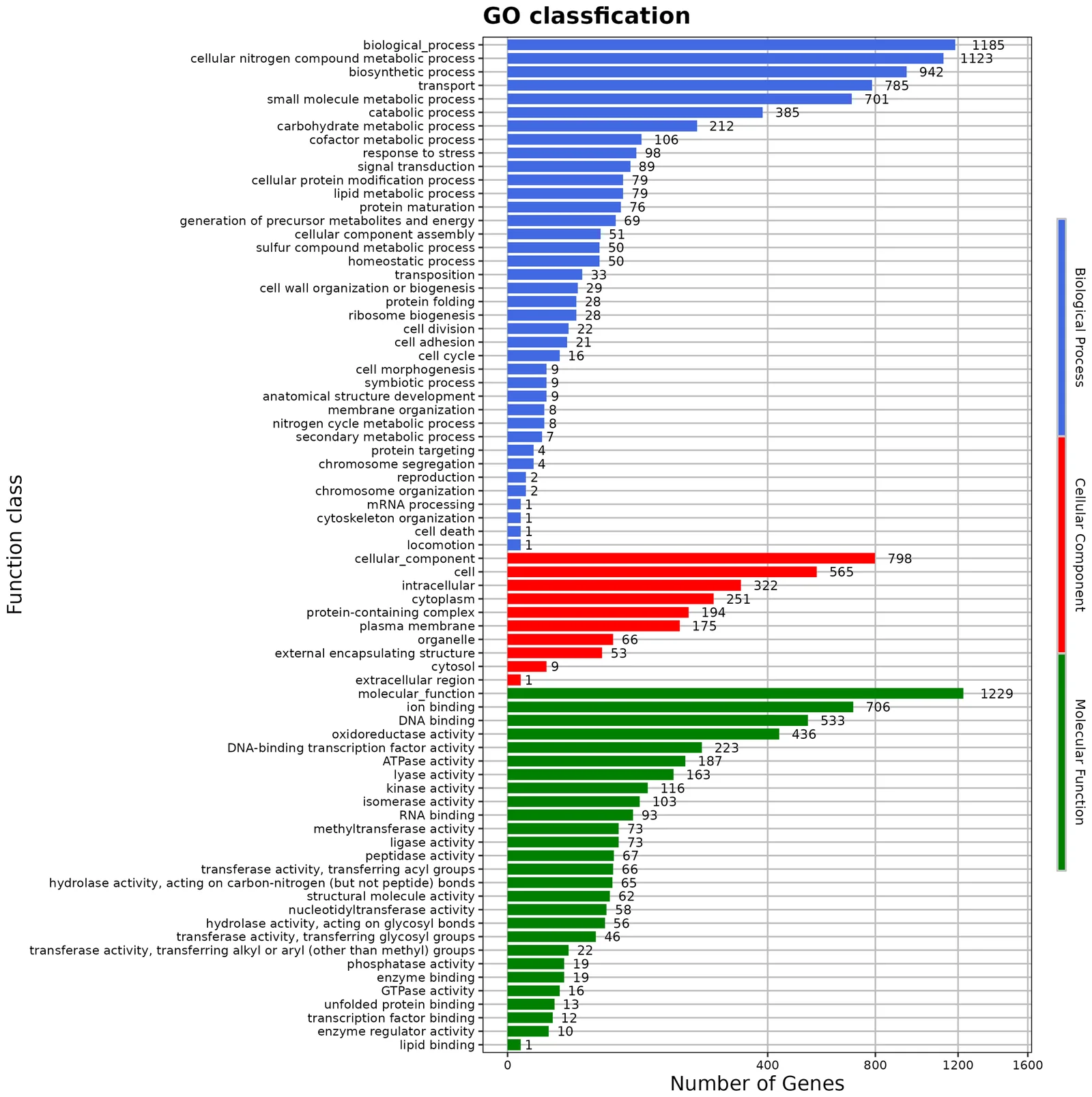
Classification statistics of GO functional annotation of *K. pneumoniae* ZYRC75. The x-axis below represents the number of genes, with the percentage of genes above. The y-axis on the left represents various categories of GO classifications. This graph illustrates the enrichment of genes in the secondary functions of GO within the context of all genes, reflecting the status of each secondary function under this background.

**Figure 8:**
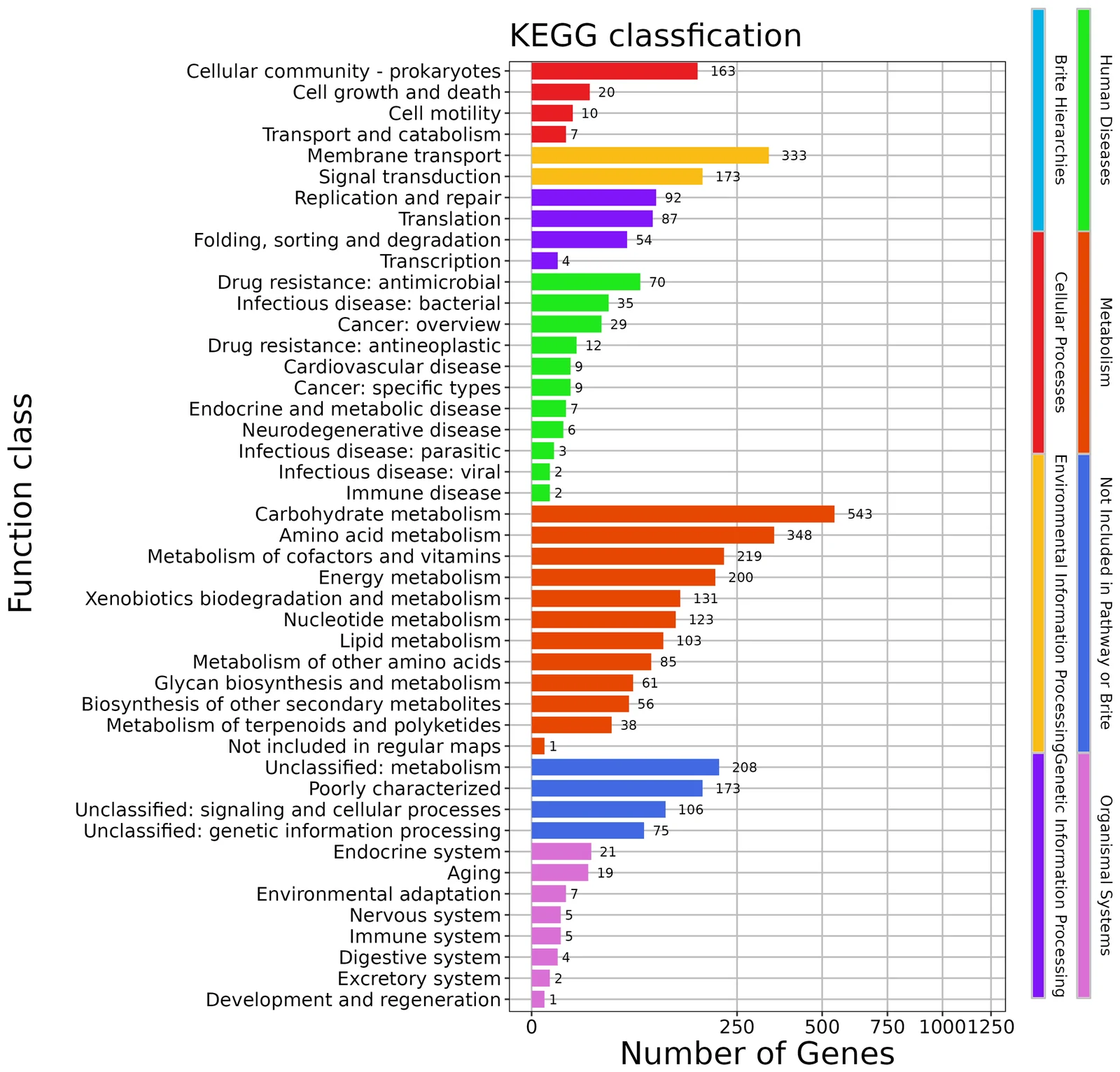
KEGG annotation classification statistics graph of *K. pneumoniae* ZYRC75. The y-axis represents KEGG secondary classifications, while the x-axis represents percentages.

The functional annotations were carried out using the GO and the KEGG databases. GO analysis indicates that there are 10 second-level categories under ‘Cellular Components’, 27 under ‘Molecular Functions’, and 38 under ‘Biological Processes’ (**Figure 7**). The KEGG pathway mapping process identified 2109 genes linked to various metabolic and cellular pathways, which are primarily grouped into three main categories: human diseases (11 pathways), metabolism (12 pathways), and genetic information processing (4 pathways) (**Figure 8**). Some genes played roles in various pathways.

## 4 Discussion

The global aquaculture industry is increasingly concerned about bacterial pathogens. As a zoonotic pathogen, *K. pneumoniae* has been recently discovered to infect a range of aquatic animals, potentially jeopardizing the sustainable development of aquaculture (Das et al., 2018). In this investigation, a pathogenic strain of *K. pneumoniae* (ZYRC75) identified as the cause of neurological disorders in bullfrogs, confirmed through a combination of bacterial Gram staining, 16S rRNA gene sequencing, physiological and biochemical properties, along with whole genome sequence and reinfection experiments.

Accurate isolation and identification of pathogenic bacteria are essential for the effective treatment of infectious diseases. This research showed that the biochemical characteristics of strain ZYRC75 were mostly aligned with those of *K. pneumoniae*. Defining the phenotypic characteristics of bacteria is a fundamental task, but association analyses based solely on these characteristics face technical hurdles and provide limited accuracy (Tindall et al., 2007). The 16S rRNA gene sequence is one of the most commonly used molecular biomarkers for bacterial identification (Busse et al., 1996). Therefore, this study employed a molecular identification method according to the 16S rRNA gene. BLAST alignment results showed that the 16S rRNA gene sequences of isolates ZYRC75 exhibited the highest resemblance to known *K. pneumoniae*. Consistent with expectations, the phylogenetic tree constructed based on 16S rRNA gene sequences revealed that strain ZYRC75 clustered with *K. pneumoniae* isolates. Based on a combination of morphological, biochemical, and phylogenetic characteristics, pathogenic strain ZYRC75 was identified as *K. pneumoniae*.

Identifying infection sources and halting transmission are crucial for effective disease control. Genotyping aids in pinpointing infection sources and transmission pathways. MLST is popular due to its high throughput, resolution, reproducibility, and ease of data sharing (Song et al., 2019). MLST is a crucial tool for the rapid and efficient investigation and classification of *K. pneumoniae* strains, reducing the risk of misclassification due to ambiguous categorization (Gorodnichev et al., 2021). This study revealed a novel sequence type for *K. pneumoniae* through the application of MLST on seven housekeeping genes.

Whole-genome sequencing is extensively utilized to examine the detailed features of bacterial pathogens (Cao et al., 2022). A number of genomes of pathogenic *K. pneumoniae* isolates isolated from humans and animals have recently been published, confirming their genomic diversity. *K. pneumoniae* strain ST11 is currently the predominant circulating clone of carbapenem-resistant *K. pneumoniae* (CRKP). Whole-genome sequencing results show that most ST11 CRKP isolates have relatively large genomes (averaging 5.65 Mb), with each isolate containing approximately 5,723 protein-coding genes, as well as 75–83 tRNA genes and 25 rRNA operons (Hou et al., 2026). The genome of ST147 *K. pneumoniae* WSF99 isolated from the wound aspiration of a diabetic cardiac patient spans 5.7Mb, with a G + C content of 56.98%, comprising 5,718 CDSs, 17 rRNAs, and 86 tRNAs (Elgayar et al., 2024). Moreover, the complete genome of *K. pneumoniae* L5–2 isolated from infant feces consists of one single chromosome (5,237,123 bp) and harbors 5,067 CDSs, 25 rRNAs and 87 tRNAs (Park et al., 2019). These human-derived *K. pneumoniae* isolates differ from the ZYRC75 isolate in terms of genomic characteristics, including genome size and the number of CDSs and RNAs. The reason is probably due to the diversity of environmental and nutritional stresses faced by different bacteria. Our findings showed that the *K. pneumoniae* ZYRC75 isolate has a single circular chromosome of 5,454,922 base pairs, with a GC content of 57.20%, containing 5,200 coding sequences, 4 rRNA genes, and 63 tRNA genes, which shared similar features of length, GC content, rRNA and tRNAs with *K. pneumoniae* 2022KP07, isolated from black-spotted frogs (Li et al., 2023). The high genomic similarity between these two isolates may be due to convergent evolutionary adaptation to the microhabitats of amphibian aquaculture. However, they also exhibit several unique genomic features that distinguish their phenotypes and adaptive capabilities. In contrast to the 2022KP07 isolate, genomic analysis identified 3 CRISPR sequences, pointing to its potential for adaptive immunity in rapidly shifting environments. The ZYRC75 isolate is characterized by 23 genomic islands, with lengths between 4,287 and 50,061 base pairs, which bestow the bacterium with special phenotypic features such as antimicrobial resistance, heightened pathogenicity, and a range of metabolic abilities, which are essential for survival and adaptation in different environmental settings. Additionally, the KEGG database analysis exposed a comprehensive hierarchical framework of biological functions particular to the ZYRC75 strain. Metabolic profiling associated 2,109 genes from the ZYRC75 isolate with specific metabolic pathways using KEGG. The primary focus of these pathways is on Signaling and Cellular Processes, Genetic Information Processing, and Metabolism. The engagement of genes in these pathways reveals ZYRC75’s robust abilities for genetic adaptation, efficient metabolic processing, and environmental response, indicating its potential for diverse biological functions and interactions in its ecological niche.

Bacterial pathogenicity is connected to the presence and expression of genes associated with virulence. Virulence factors such as capsule polysaccharide, fimbriae, and outer membrane proteins in *K. pneumoniae* facilitate evasion of host immune defenses, leading to cell death post-infection (Martin and Bachman, 2018). Pathogenic strains of *K. pneumoniae* are generally characterized by the presence of various virulence factors. For instance, the *K. pneumoniae* strain KPY01, isolated from the spotted catfish (*Ophichthys oculatus*), has been identified to harbor the virulence-associated gene *wabG*, *uge*, *mrkD*, *ureA* and *fimH* (Wu et al., 2021). The pathogenicity of highly virulent *K. pneumoniae* strains isolated from patient urine is multifaceted, largely due to the presence of capsular virulence genes such as *rmpA*, *kfu*, *fimH*, *mrkD*, *allS*, *iutA*, *magA*, *entB* and *ybtS* (Rastegar et al., 2019). In this study, whole-genome sequencing revealed the presence of 179 virulence genes, which may be associated with biological processes, including the presence of specific virulence-associated factors such as *mrkD*, *fimH*, *fimA*, *fimB*, *ompA*, *entC*, *entS*, *ecpB*, *gspG*, *impJ*, *iroE*. A bacterium is considered highly virulent if its LD_50_ is less than 1.0 × 10^5^ CFU/animal (Sun et al., 2025). A virulence assay conducted via intramuscular injection demonstrated that the LD_50_ of *K. pneumoniae* ZYRC75 was 2.51 × 10^3^ CFU/bullfrog, suggesting that the isolate exhibits significant virulence against bullfrogs. Infections with *K. pneumoniae* in bullfrogs lead to multi-organ damage and congestive edema, similar to the effects of *E. miricola* and *A. hydrophila* infections. Consequently, anatomical analyses might cause an incorrect diagnosis of the infection. Previous studies showed that bullfrogs infected by *K. pneumoniae* did not display torticollis and rotational swimming, as observed in *E. miricola* infections (Lin et al., 2023). However, bullfrogs challenged with the isolated ZYRC75 exhibits significant symptoms of neurologic disorders, which should be further studied.

Antibiotic resistance is a major global public health threat, with resistance genes significantly risking genetic pollution in aquatic environments (Pruden et al., 2006; Tian et al., 2023). Prior research has demonstrated that *K. pneumoniae* strain NW202109 exhibits resistance to macrolides, glycopeptides, β-lactams, carbapenems and neomycin (Lin et al., 2023). Similarly, Wu et al. documented resistance in *K. pneumoniae* strain KPY01 to amoxicillin, rifampicin, gentamicin and erythromycin (Wu et al., 2021). In this study, antimicrobial susceptibility testing showed that the ZYRC75 strain exhibited multi-antimicrobial resistance, particularly against sulfonamides, carbapenems, dihydrofolate reductase inhibitors, tetracyclines, rifamycins, lincosamides, nitrofurans, macrolides, quinolones, cephalosporins, aminoglycosides, penicillins and chloramphenicols. The isolate showed intermediate susceptibility to the nitrofuran derivative nitrofurantoin and the sulfonamide derivative sulfamethoxazole, while being sensitive only to polymyxins (polymyxin B). Therefore, the choice of antibiotics to treat neurologic disorders caused by *K. pneumoniae* in cultured bullfrogs is limited. Our study indicates that the *K. pneumoniae* ZYRC75 isolate exhibits significant resistance to various antibiotics, consistent with earlier findings in black-spotted frog isolates. These observations underscore the substantial resistance that *K. pneumoniae* has developed against commercially available antimicrobial agents utilized in aquaculture, highlighting the urgent necessity of identifying alternative therapeutic strategies to effectively mitigate its dissemination. Antibiotic resistance genes can be horizontally transferred among strains and species through mechanisms such as plasmid conjugation, phage transduction, and extracellular DNA transformation (Haaber et al., 2017). Previous studies have shown that antibiotic resistance genes are transferred horizontally between *K. pneumoniae and Escherichia coli* (Haverkate et al., 2015), as well as among species of the *Acinetobacter* and *Elizabethkingia genera* (Fu et al., 2021). In this study, whole-genome sequencing of *K. pneumoniae* revealed a substantial number of predicted antibiotic resistance genes, underscoring the potential risk of antibiotic resistance dissemination.

## 5 Conclusion

In summary, a new MLST-based sequence types *K. pneumoniae* ZYRC75 was isolated from diseased bullfrogs and characterized. The isolate exhibited high pathogenicity, causing neurologic disorders in bullfrogs and posing a significant threat to bullfrogs. This study also conducted antimicrobial susceptibility testing on the isolate. Whole genome analysis revealed that this strain is highly virulent and multidrug-resistant, with 179 virulence genes and 157 drug resistance genes. These findings provide a comprehensive genomic and phenotypic framework for ZYRC75, offering valuable insights for future research into the mechanisms of antimicrobial resistance and pathogenicity in *K. pneumoniae*.

## Data Availability

The datasets presented in this study can be found in online repositories. The names of the repository/repositories and accession number(s) can be found in the article/Supplementary Material.
